# Advanced Estimation Techniques for Vehicle System Dynamic State: A Survey

**DOI:** 10.3390/s19194289

**Published:** 2019-10-03

**Authors:** Xianjian Jin, Guodong Yin, Nan Chen

**Affiliations:** 1 School of Mechatronic Engineering and Automation, Shanghai University, Shanghai 200072, China; 2 State Key Laboratory of Automotive Simulation and Control, Jilin University, Changchun 130025, China; 3 School of Mechanical Engineering, Southeast University, Nanjing 211189, China

**Keywords:** vehicle dynamics, vehicle state estimation, model-based approach, data-driven-based approach

## Abstract

In order to improve handling stability performance and active safety of a ground vehicle, a large number of advanced vehicle dynamics control systems—such as the direct yaw control system and active front steering system, and in particular the advanced driver assistance systems—towards connected and automated driving vehicles have recently been developed and applied. However, the practical effects and potential performance of vehicle active safety dynamics control systems heavily depend on real-time knowledge of fundamental vehicle state information, which is difficult to measure directly in a standard car because of both technical and economic reasons. This paper presents a comprehensive technical survey of the development and recent research advances in vehicle system dynamic state estimation. Different aspects of estimation strategies and methodologies in recent literature are classified into two main categories—the model-based estimation approach and the data-driven-based estimation approach. Each category is further divided into several sub-categories from the perspectives of estimation-oriented vehicle models, estimations, sensor configurations, and involved estimation techniques. The principal features of the most popular methodologies are summarized, and the pros and cons of these methodologies are also highlighted and discussed. Finally, future research directions in this field are provided.

## 1. Introduction

To improve ground vehicle handling stability and passenger safety, a large number of advanced vehicle-active safety dynamic control systems, such as the direct yaw control system (DYC) [1,2,3,4], anti-lock braking systems (ABS) [5,6], four-wheel steering system (4WS) [7,8], active front steering system (AFS) [2,9], active suspension system (ASS) [9,10], adaptive cruise control (ACC) [11], collision avoidance system (CAS) [12], and other advanced driver assistance systems (ADAS) towards a connected and automated driving vehicle have been developed and brought into the market in recent years [13,14,15].

However, the practical effects and potential performance in vehicle-active safety dynamic control systems depend strongly on real-time knowledge of vehicle states. For instance, accurate knowledge about longitudinal and lateral tire-road forces and vehicle sideslip angle means a better prediction of the real-time road condition and the potential vehicle trajectories, leading to better vehicle motion management. Unfortunately, some fundamental vehicle states are difficult to measure directly in a standard car due to both technical and economic reasons, wherein additional vehicle sensors are too expensive and measured signals may be lost under complicated driving environments [16,17,18,19,20]. As a consequence, these important vehicle states and parameter information (e.g., vehicle velocity, vehicle sideslip angle, tire-road interactive force, vehicle mass, etc.) must be estimated or observed.

Vehicle system dynamic state estimation has been widely discussed and investigated in the literature. Different vehicle estimations can be further classified into two categories depending on whether the vehicle model is required. The first category is the model-based estimation approach, whereas the second category is the data-driven-based estimation approach. Vehicle model-based estimation approaches can be further divided into two sub-categories: the vehicle kinematic model-based estimation approach and the vehicle dynamic model-based estimation approach. In contrast to the kinematic model-based estimation approach that attempted to estimate vehicle system state by finding the direct kinematic correlations between measurements and estimated states, the vehicle dynamic model-based estimation technique utilizes mathematical models to describe the transient behavior in vehicle system dynamics so that it possesses higher state estimation accuracy, continuously attracting the increased interest of academics. Different types of vehicle dynamic model-based approaches, along with different estimation strategies, have been developed and studied in recent years. More recently, with the rapid development of artificial intelligence, the data-driven-based estimation approach, artificial neural network (ANN)-based estimations in particular, have shown promising perspectives in vehicle state estimation applications.

**Figure 1:**
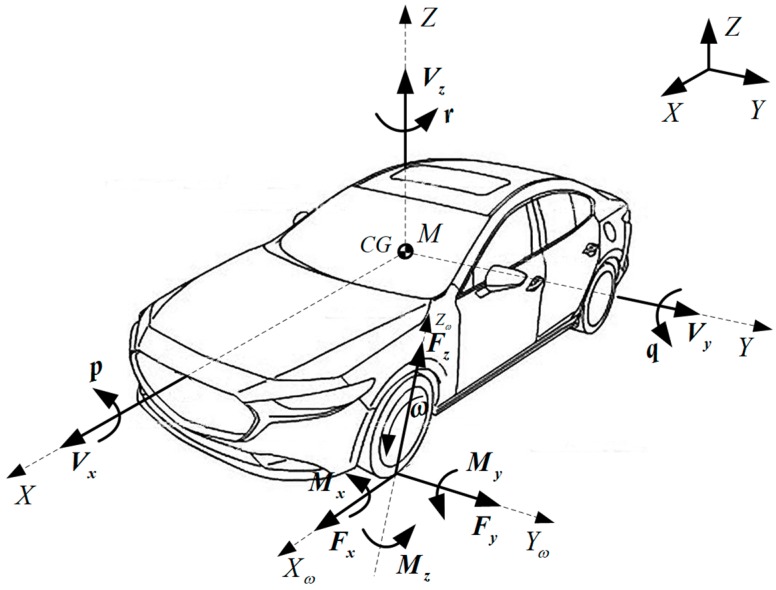
Illustration of fundamental vehicle states and parameters.

Various published estimation studies are related to fundamental vehicle states that are unmeasured directly, and these estimated states (shown in Figure 1) mainly include vehicle operating states (e.g., vehicle lateral and longitudinal velocities ***V_y_***, ***V_x_***; vehicle sideslip angle *β* at center of gravity (CG); etc.) and tire–road interaction (e.g., tire–road lateral and longitudinal forces ***F_y_***, ***F_x_***; tire longitudinal stiffness *C_s_* and cornering stiffness *C_α_*; tire–road friction coefficient (TRFC) *µ*; tire vertical load ***F_z_***; etc.). Meanwhile, ***r***, ***q***, and ***p*** are yaw rate, pitch rate, and roll rate, respectively; ***ω*** stands for angular velocity of the wheel; and ***M_x_***, ***M_y_***, and ***M_z_*** are the overturing moment, wheel torque, and aligning moment of the tire, respectively. 

Thereby, this paper presents a technical survey for development and recent research progress of fundamental vehicle system dynamic state estimation in terms of vehicle models, estimations, sensor configurations, and advanced estimation methodologies. The remainder of the paper is organized as follows: In Section 2, model-based estimation approaches are analyzed and reviewed. In Section 3, data-driven-based estimation approaches for vehicle dynamic state are introduced and discussed. Finally, conclusions are offered in Section 4.

## 2. Model-Based Vehicle State Estimation

As mentioned above, vehicle model-based estimation consists of vehicle kinematic model-based estimation approach and vehicle dynamic model-based estimation approach. Vehicle kinematic model-based estimation is to estimate vehicle dynamic states on the basis of the kinematic relationship between estimated states and the vehicle-measured data. Several studies relating to this aspect can be found [16,17,18,19,20]—for instance, in [16], the direct relationship among derivatives about vehicle lateral velocity, yaw rate, and the lateral acceleration at CG is considered, and then vehicle lateral velocity is estimated with the integration method

(1) $$V_{y}=\int {\dot{V}}_{y}=\int \left(-V_{x}r_{z}+a_{y}\right)dt.$$

As presented in [17], vehicle sideslip angle *β* can be estimated by

(2) $${\hat{\beta }}_{kin}=\int {\dot{\hat{\beta }}}_{kin}dt=\int \left(\frac{a_{y}+g\phi }{V_{x}}-r_{z}\right)dt.$$

The kinematics-based estimation is a direct integration method of vehicle state estimation that also integrates the noise signal when integrating the useful sensor signals, whereas the vehicle sensor has a fixed calibration error and drift error wherein the accumulated error will increase continuously after a long time integration, and finally the estimation result will be seriously distorted and the estimated vehicle states will thus be extremely inaccurate. Considering the limitations of the kinematics-based estimation approaches, the majority of studies tend to research vehicle dynamic model-based estimation that applies physical and mathematical models to capture the inherent nature of vehicle system dynamics to estimate vehicle state. Different vehicle dynamics models may be selected for different estimation applications. Here, typical vehicle estimation models including the vehicle dynamics model and tire model are introduced.

### 2.1. Vehicle Dynamics Estimation Model

#### 2.1.1. Vehicle Dynamics Model

**Figure 2:**
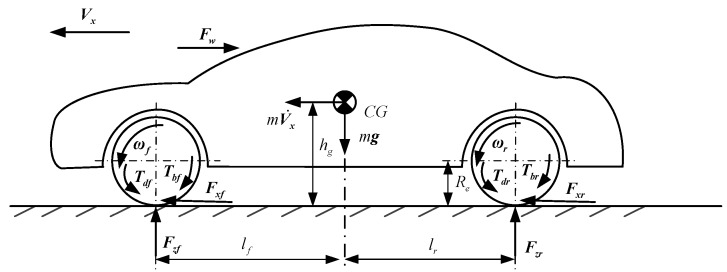
Two-wheel model of vehicle longitudinal dynamics.

*1) Longitudinal model*. The vehicle longitudinal model shown in Figure 2 is applied to describe the vehicle longitudinal dynamics of braking and driving maneuvers. When only the vehicle longitudinal motion is considered, along with the road grade angle, the lateral and other vehicle movements are negligible. The left and right wheels of a vehicle can be combined into a wheel by ignoring the difference in motion between the left and right wheels. Then, a vehicle longitudinal model called the longitudinal two-wheel model, consisting of the vehicle longitudinal dynamics model and the rotational dynamics of the wheel, can be described as [3,6,12]

(3) $$m{\dot{V}}_{x}=F_{\mathrm{xf}}+F_{\mathrm{xr}}-F_{w}-F_{f},$$

(4) $$I_{\omega i}{\dot{\omega }}_{i}=T_{\mathrm{di}}-T_{\mathrm{bi}}-R_{e}F_{\mathrm{xi}},$$

(5) $$F_{w}=C_{d}\rho A_{f}V_{x}^{2}/2,\;F_{f}=\mu mg,$$

where *m* is the vehicle mass; ***F_xf_***, ***F_xr_*** are the longitudinal tire force of front and rear axles, respectively; ***F_w_***, ***F_f_*** are the aerodynamic drag force and the rolling resistance force, respectively; *C_d_* is an aerodynamic drag coefficient; *ρ, A_f_* are air density and windward area of the vehicle, respectively; ***T_di_**,* ***T_bi_*** stand for the driving and braking torque and of the specific wheel, respectively; ***ω_i_***, *I_ωi_* are the angular velocity of the wheel and moment of inertia of the wheel, respectively; and *R_e_* is the effective radius of the tire. Subscripts *i* = *f*,*r* stand for the front and rear, respectively.

*2) Single-track model*. Different from the longitudinal model, the main application of the single-track model (also called the bicycle model) is to estimate vehicle lateral states. By considering lateral and yaw motions, here other vehicle motions such as longitudinal, pitch, roll, and vertical motions are neglected, and then the vehicle lateral dynamics equations of the widely used single-track model can be presented as [21,22,23,24,25,26,27,28,29,30,31,32]

(6) $$mV_{x}(\dot{\beta }+r_{z})=F_{\mathrm{yr}}+F_{\mathrm{yf}}\cos\delta_{f}+F_{\mathrm{xf}}\sin\delta_{f},$$

(7) $$I_{zz}{\dot{r}}_{z}=F_{\mathrm{yf}}\cos\delta_{f}l_{f}-F_{\mathrm{yr}}l_{r},$$

where *I_zz_* is the yaw moment of inertia; *l_f_* and *l_r_* are the distances from the front and rear axle to the CG, respectively; *δ_f_* is the front steering angle and it also assumes that steering action only occurs in the front wheel; and ***F_yf_***, ***F_yr_*** are the lateral tire force of front and rear wheels, respectively.

**Figure 3:**
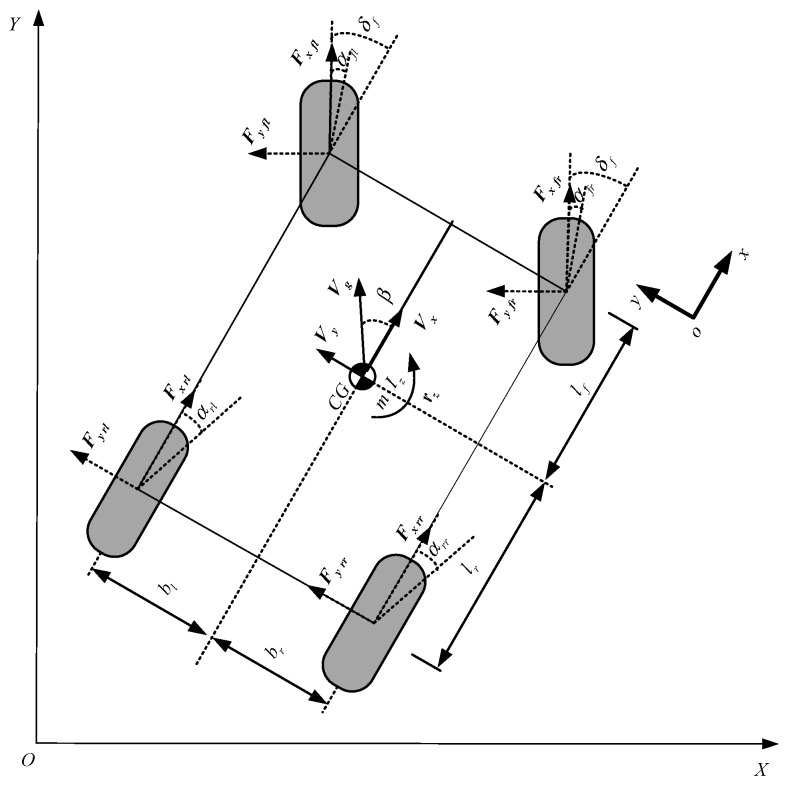
Double-track model of vehicle lateral dynamics.

*3) Double-track model*. Assume that the vehicle movement is planar motion, and other vehicle motions such as pitch, roll, and vertical motions are also neglected. When the longitudinal motion is added and the dynamics for four wheels are also addressed for the single-track model, causing the four wheel vehicle model to then involve longitudinal and lateral motions and yaw motion, the so-called double-track model (shown in Figure 3) is often used to estimate longitudinal and lateral states, being modeled as [27,33,34]. 

Longitudinal motion:

(8) $$\begin{array}{ll} m({\dot{V}}_{x}-V_{y}r_{z})= & (F_{\mathrm{xfl}}+F_{\mathrm{xfr}})\cos\delta_{f}-(F_{\mathrm{xfl}}+F_{\mathrm{xfr}})\sin\delta_{f}\\ & +F_{\mathrm{xrl}}+F_{\mathrm{xrr}}-C_{d}\rho A_{f}V_{x}^{2}/2-\mu mg \end{array}$$

$${\dot{V}}_{x}=a_{x}+V_{y}r_{z}.$$

Lateral motion:

(9) $$\begin{array}{c} m({\dot{V}}_{y}+V_{x}r_{z})=(F_{\mathrm{yfl}}+F_{\mathrm{yfr}})\cos\delta_{f}+(F_{\mathrm{yrl}}\\ +F_{\mathrm{yrr}})+(F_{\mathrm{yfl}}+F_{\mathrm{yrl}})\sin\delta_{f} \end{array}$$

$${\dot{V}}_{y}=a_{y}-V_{x}r_{z}.$$

Yaw motion:

(10) $$\begin{array}{ll} I_{zz}{\dot{r}}_{z}= & (F_{\mathrm{yfl}}\sin\delta_{f}-F_{\mathrm{xfl}}\cos\delta_{f}+F_{\mathrm{xfr}}\cos\delta_{f}-F_{\mathrm{yfr}}\sin\delta_{f})b_{l}\\ & -(F_{\mathrm{xfl}}\sin\delta_{f}+F_{\mathrm{yfl}}\cos\delta_{f}+F_{\mathrm{xfr}}\sin\delta_{f}+F_{\mathrm{yfr}}\cos\delta_{f})l_{f}\\ & +(F_{\mathrm{xrr}}-F_{\mathrm{xrl}})b_{r}+(F_{\mathrm{yrr}}+F_{\mathrm{yrl}})l_{r} \end{array}$$

In the above dynamics equations, *δ_fl_* and *δ_fr_* are the front steering angles of the left and right wheel, respectively, and *I_zz_* and *M_z_* stand for the vehicle moment of inertia and yaw moment, respectively.

**Figure 4:**
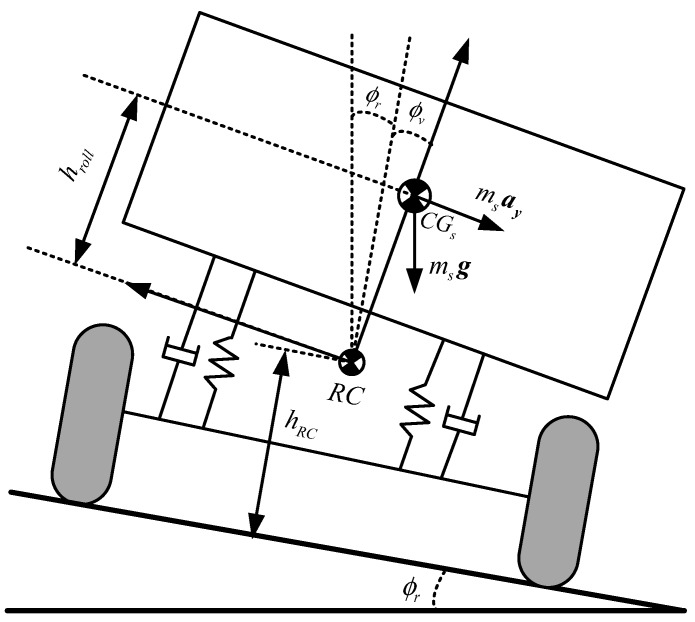
Vehicle roll dynamics model with road bank angle.

*4) Roll dynamics model*. The vehicle roll dynamics model shown in Figure 4 is introduced to estimate roll states. When vehicle roll motion is considered in steering maneuvers, and the following assumptions are made, lateral and vertical motions of RC are therein ignored, and then the location of the roll axis is fixed with constant height *h_RC_*. Bouncing and pitching movements of sprung mass are not considered, and roll angle is relatively small. The vehicle roll dynamics model considering the road bank angle can be established, and it can be derived from the roll moment balance [14,22,31,33,34].

(11) $$I_{xx}{\ddot{\phi }}_{v}=m_{s}h_{roll}(V_{y}+V_{x}\dot{\varphi })+m_{s}h_{roll}g(\phi_{v}+\phi_{r})-C_{\phi }{\dot{\phi }}_{v}-K_{\phi }\phi_{v}$$

where *K_φ_* and *C_φ_* are the roll stiffness and roll damping coefficient, respectively; *m_s_*, *I_xx_* are sprung mass and sprung mass moment of inertia about roll axes, respectively; *h_roll_* is the distance from roll axis to the CG of sprung mass; *φ*, *φ_v_*, and *φ_r_* are the yaw angle, the roll angle, and the road bank angle, respectively.

#### 2.1.2. Tire Dynamics Model

*1) LuGre tire model*. When the vehicle only keeps longitudinal motion, the LuGre tire model can be used to represent tire longitudinal force for the longitudinal two-wheel model under longitudinal friction condition [1,3,5,6,12]. The LuGre model incorporates transient behaviors of friction and road conditions by assuming an internal dynamic state of friction between tire and road interaction, which can be described as

(12) $$\begin{array}{l} F_{xi}=\mu F_{zi}\\ \mu =(\sigma_{0}z_{f}+\sigma_{1}{\dot{z}}_{f}-\sigma_{2}v_{r}) \end{array}$$

where

$$\begin{array}{l} {\dot{z}}_{f}=-v_{r}-\frac{\sigma_{0}\left|v_{r}\right|}{f(v_{r})}\rho z_{f}\\ f(v_{r})=\mu_{c}+(\mu_{s}-\mu_{c})e^{-{\left|v_{r}/v_{s}\right|}^{1/2}}\\ v_{r}=R_{e}\omega -V_{x} \end{array}$$

where ***F_xi_***, ***F_zi_*** represent the longitudinal and vertical tire forces of the wheel, respectively. Other relevant parameters of the LuGre tire model can be found in [1,3,5,6,12].

*2) Linear tire model*. When the tire slip angles and slip ratios tend to be small, longitudinal and lateral tire forces can then be linearly approximated by [21,22,23,24,25,26,27]

(13) $$\begin{array}{l} F_{xij}=C_{sij}s_{ij}\\ F_{yij}=C_{\alpha ij}\alpha_{ij} \end{array}$$

where *C_sij_* and *C_αij_* are tire longitudinal stiffness and cornering stiffness, respectively; and *s_ij_* and *α_ij_* are tire slip ratio and the tire slip angle, respectively. Simultaneously, the tire slip angles can be given as

(14) $$\begin{array}{l} \alpha_{f}=\delta_{f}-l_{f}r_{z}/V_{x}-\beta \\ \alpha_{r}=l_{f}r_{z}/V_{x}-\beta \end{array}$$

It is worth noting that the linear tire model is appropriate under normal driving conditions, whereas when a vehicle experiences extreme handlings where the tire operates in the nonlinear region, the widely used nonlinear tire models including the Pacejka model, Dugoff model, and Brush model need to be introduced and used.

*3) Pacejka tire model*. The Pacejka tire model “Magic Formula” uses the same set of trigonometric function formulae to uniformly express the longitudinal and lateral forces of the tire. The tire–road forces with the nonlinear Pacejka tire model [28,30,32,33,34] are modeled as

(15) $$\left\{ \begin{array}{l} Y(x)=y(x)+S_{v}\\ y=D\sin(C\arctan(Bx-E(Bx-\arctan Bx)))\\ x=X+S_{h} \end{array}\right.$$

where *Y* stands for the lateral tire–road force ***F_y_*** or longitudinal tire–road force ***F_x_***, and *X* stands for the tire slip angles *α* or slip rates *s*. Then longitudinal and lateral tire forces can be presented as

(16) $$F_{xij}=D_{ij}\sin\left[C_{ij}\tan^{-1}\left\{B_{ij}\left(1-E_{ij}\right)s_{ij}+E_{ij}\tan^{-1}\left(B_{ij}s_{ij}\right)\right\}\right],$$

(17) $$F_{yij}=D_{ij}\sin\left[C_{ij}\tan^{-1}\left\{B_{ij}\left(1-E_{ij}\right)\alpha_{ij}+E_{ij}\tan^{-1}\left(B_{ij}\alpha_{ij}\right)\right\}\right],$$

where tire parameters *B*, *C*, *D,* and *E* can be determined on the basis of tire vertical force and tire–road friction coefficient [28,30,32,33,34]. 

*4) Dugoff tire model*. The Dugoff nonlinear tire model synthesizes the tire property into the parameters of the tire longitudinal stiffness and the tire cornering stiffness, which refers to certain tire–specific parameters. Longitudinal and lateral tire–road forces can be defined as follows [31,35]:

(18) $$F_{xij}=\frac{C_{sij}s_{ij}}{1-s_{ij}}f(S),$$

(19) $$F_{yij}=\frac{C_{\alpha ij}\tan\alpha_{ij}}{1-s_{ij}}f(S),$$

where

$$S=\frac{\mu F_{zij}(1-\varepsilon_{r}V_{x}\sqrt{s_{ij}^{2}+\tan^{2}\alpha_{ij}})}{2\sqrt{C_{sij}^{2}s_{ij}^{2}+C_{\alpha ij}^{2}\tan^{2}\alpha_{ij}}}(1-s_{ij}^{2})$$

$$f(S)=\left\{ \begin{array}{ll} 1 & S>1\\ S(2-S) & S<1 \end{array}\right.$$

where *ε_f_* and *ε_r_* are the front and rear roll steer coefficients, respectively.

*5) Brush tire model*. The Brush tire model can also reflect nonlinear characteristics of combined longitudinal and lateral tire force under the friction ellipse. The tire dynamics in the Brush tire model can be written as [2]

(20) $$F_{xij}=\frac{C_{si}f(\xi_{i})s_{i}}{\xi_{i}(1+s_{i})},$$

(21) $$F_{yij}=\frac{C_{\alpha i}f(\xi_{i})\tan\alpha_{i}}{\xi_{i}(1+s_{i})},$$

where

$$\begin{array}{l} f(\xi_{i})=\{\begin{array}{cc} \xi_{i}-\frac{1}{3\mu F_{zi}}\xi_{i}^{2}+\frac{1}{27\mu^{2}F_{zi}^{2}}\xi_{i}^{3} & if\;\xi_{i}\leq 3\mu F_{zi}\\ \mu F_{zi} & else \end{array}\\ \xi_{i}=\sqrt{C_{si}^{2}{\left(\frac{s_{i}}{1+s_{i}}\right)}^{2}+C_{\alpha i}^{2}{\left(\frac{\tan\alpha_{i}}{1+s_{i}}\right)}^{2}}\\ s_{i}=\frac{R_{e}\omega_{i}-V_{x}}{\max(R_{e}\omega_{i},V_{x})} \end{array}$$

and subscript *i* stands front *f* and rear *r*, respectively.

**Figure 5:**
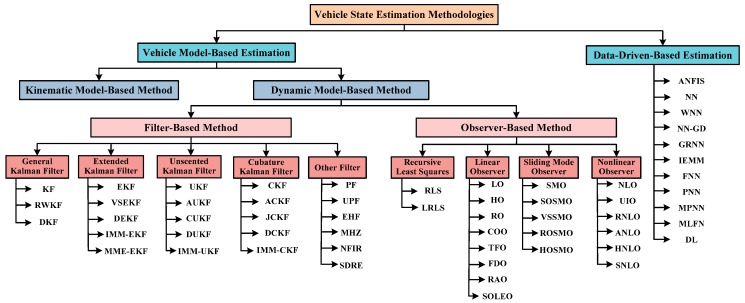
Categorization of vehicle dynamic state estimation methodologies.

On the basis of these vehicle dynamics models and tire models, various estimation strategies and approaches are introduced in this section. The vehicle dynamic model-based estimation approach is further classified into two main categories consisting of filter-based vehicle state estimation and observer-based vehicle state estimation. Filter-based vehicle state estimation is further divided into several sub-categories such as general Kalman filter (KF), extended Kalman filter (EKF), unscented Kalman filter (UKF), cubature Kalman filter (CKF), and other filter. Observer-based vehicle state estimation can also be categorized as recursive least squares method (RLS), linear observer, sliding mode observer, and nonlinear observer. Categorization of vehicle dynamic state estimation methodologies is shown in Figure 5. The most popular methodologies of the two main categories from the perspective of methodologies, models, estimations, and sensor configurations for vehicle state estimation are respectively summarized in Table 1 shown in Section 2.2 and Table 2 shown in Section 2.3.

### 2.2. Filter-Based Vehicle State Estimation

#### 2.2.1. Kalman Filter(KF)-Based Estimation

*1) General Kalman filter(KF)-based estimation*. On the basis of the general KF framework, the stochastic estimation KF in [21] derived from the two degrees-of-freedom (DOF) bicycle model with the linear tire model was proposed to estimate the vehicle lateral velocity. Utilizing the combination of global positioning system (GPS) and other sensor signals, the estimated accuracy of vehicle states can be improved [22,23,24,25,26]. In [22], a novel KF estimation method with roll dynamics is studied to estimate vehicle roll angle and vehicle sideslip angle using real-time lateral tire force measured from multisensing hub (MSHub) units as an external input, and field tests with experimental in-wheel-motor-driven electric vehicles (IWM-EV) evaluated the effectiveness and accuracy of the developed estimator. The work [23] developed KF-based fusion technology of multi-sensor fusing from GPS and inertial navigation system (IMU) measurements to estimate vehicle sideslip angle through GPS and IMU complementing each other, whereas the study [25] employed the grade inertial sensor and a two-antenna GPS system to estimate longitudinal velocity, sideslip angle, and other lateral states. In [26], the dual Kalman filter (DKF) technique with GPS measurements was introduced for identification of tire cornering stiffness, and its experiments on both flat and banked curve roads validated the effectiveness of the identification method. A random-walk Kalman filter (RWKF) in [27] was presented for the lateral and longitudinal tire-force estimation that consisted of vertical tire-force estimation, shaft torque estimation, and combined tire-force estimation.

Note that the 2-DOF bicycle model with linear tire model-based KF estimation is valid for small tire slip angles, whereas vehicle dynamic and tire dynamics possess high nonlinearities when the vehicle undergoes high accelerations under extreme handling conditions. This means that the linear model-based KF estimator is not sufficiently reliable for all operational maneuvers. To face the requirement and challenge of system nonlinearities in vehicle dynamics estimation, different nonlinear Kalman filter estimation methods, such as the extended Kalman filter (EKF), unscented Kalman filter (UKF), and cubature Kalman filter (CKF), have been developed.

*2) Extended Kalman filter (EKF)-based estimation*. By using the single-track model coupling with a simplified Pacejka model identified as *FTyre_y,i_*, which can reduce the need of tire parameters and computational load in estimation processes, the authors introduce EKF to vehicle slip angle estimation in [28].

(22) $${\dot{F}}_{y,i}=\frac{V_{x}}{d_{i}}(FTyre_{y,i}(\alpha_{i},F_{z},i;\xi )+F_{y,i}).$$

In [29], on the basis of the first-order tire dynamics model that aimed at enhancing transient behavior of tires developed from the linearized tire model and relaxation time constant, EKF was designed to estimate the vehicle sideslip angle and tire cornering stiffness, and its effectiveness was evaluated and compared with kinematics-based method through field tests on IWM-EV.

(23) $$\tau_{lag,i}{\dot{F}}_{yi}+F_{yi}={\bar{F}}_{yi}$$

where *τ_lag,i_* is relaxation time constant related to ***V_x_*** and tire relaxation length. The steering torque obtained from the simplified EPS instead of steering angle is used in [30] to estimate vehicle sideslip angle because of the fact that steering torque has a more rapid response when compared to steering angle. The effects of various vehicle–road system models for EKF estimation of vehicle lateral states and lateral tire force were investigated in [33,34], which consisted of vehicle models including 2-DOF, 3-DOF, and 4-DOF, as well as tire models involving linear tire and Pacejka tire. Utilizing the double-track vehicle model and Dugoff tire model, the vehicle velocities, as well as tire lateral forces, were estimated and reconstructed [35]. The work [36] discussed vehicle slip angle estimation when the vehicle lay inside an instability region under extreme steering maneuvers, and a variable structure EKF (VSEKF) was brought forward to estimate the vehicle slip angle via the modified Dugoff tire model and vehicle model where load transfer effect is compensated and integrated into the model. Two principal blocks applying two EKFs in [37] were designed—one that estimated vertical tire forces considering lateral load transfer and the other that estimated lateral tire–road forces. The research covered in [38] was a novel scheme to improve the computational performance of vehicle lateral and longitudinal velocities of EKF estimator through field programmable gate array (FPGA) and system on programmable chip (SoPC). The minimum model error (MME) criterion-based EKF estimation method was developed for two-motor-driven vehicles in [39] to obtain lateral and longitudinal velocities and lateral and longitudinal tire forces by eliminating the estimator error caused from the nonlinear vehicle modeling error. In [40], a new EKF estimation process was proposed in order to estimate vehicle sideslip angle, lateral tire forces, and TRFC by combining the single-track vehicle model and Burckhardt/Kiencke adaptive tire model that takes into account variations in road friction, evaluating it with two nonadaptive tire-based EKF estimators. An EKF method derived from the Pacejka tire model or modified Pacejka tire model has been proposed to estimate vehicle lateral states, tire-road forces, and TRFC [41,42,43,44]. By equipping additional GPS sensors [45,46,47], an EKF-based fusion methodology integrating in-vehicle sensors and single-frequency double-antenna GPS was developed in [46] to obtain reliable estimation about vehicle state information, such as vehicle sideslip and roll angle, while EKF estimation in [47] considered the vehicle sideslip angle and TRFC by fusing measurements of GPS and IMU. In [48], the EKF with parameter adaption was investigated to estimate vehicle sideslip angle and cornering stiffness; 262 test drives validated that the estimator can deal with banked corners and varying friction coefficients.

In order to simultaneously estimate vehicle states and parameters, the dual extended Kalman filter (DEKF) technique was proposed [43,49,50,51]. The work [49] first proposed a DEKF technique with two KFs in parallel, splitting the state and parameter estimation problems, as well as the feasibility and advantages of the DEKF estimator for combined vehicle states and parameter estimation, such as vehicle mass and yaw moment of inertia at CG, which are demonstrated in the theory. The DEKF-based state and parameter estimation for articulated heavy vehicles was discussed in [50], wherein vehicle states and parameters including height of trailer mass and roll moment of inertia were estimated and implemented through experimental tests. DEKF was also applied to estimate vehicle states and TRFC in [51].

To adapt the complex estimation environment from various vehicle driving conditions, the interacting multiple model (IMM) approach has been developed to estimate vehicle dynamic states [52,53,54,80]. The research in [52] reported IMM-EKF estimation approach of vehicle states and road conditions, wherein the system model was constructed and modeled via 10 system models considering tire nonlinearity and different road friction conditions; the system model can be switched among 10 system models in a probabilistic manner so that states and TRFC can be estimated with high accuracy. In [53], utilizing real-time measurements from in-vehicle sensors of in-wheel motor-driven electric vehicles, the IMM estimation method was designed to estimate vehicle sideslip and roll angle, as well as lateral tire–road forces, by integrating two kinds of different vehicle–road system models to adapt variable driving conditions. The results show improved estimation accuracy of vehicle-dynamic parameters and vehicle–road interaction compared with the conventional approach.

*3) Unscented Kalman filter (UKF)-based estimation*. In contrast to the EKF method, UKF utilizes a set of sigma points to realize nonlinear transformation, which acts directly on the nonlinear vehicle dynamics systems to approximate the states. In [55], an unscented Kalman filter (UKF) method making full use of driving torques from a four-wheel-drive hybrid vehicle was employed to estimate vehicle velocities on the basis of the UniTire model in different driving modes, and the UKF-based vehicle sideslip angle was obtained as valued information of lateral stability control for in-wheel motored electric vehicles [56]. Considering the effect of model non-linearity, uncertainty, and road friction conditions, an adaptive variable structural UKF (AUKF) was studied in [57] to compensate the model uncertainty for vehicle sideslip angle estimation. The vehicle state estimation with AUKF addressed in [58] was a practical road influence of noise variance and covariance on the estimation accuracy of UKF, whereas the proposed constrained UKF (CUKF) technique in [59] fully took state boundaries, measurement noise, and nonlinearities in to account to prevent unphysical vehicle sideslip angle estimation. To address vehicle system un-modeled dynamics and nonlinearities, EKF and UKF techniques for vehicle sideslip angle and tire–road forces estimation in [60] were proposed and compared, and road results demonstrated that estimation performances of UKF were far better than EKF with respect to road variation, which was tested from an experimental car equipped with noncontact optical correvits. A hybrid UKF estimator connected with two sub-estimators consisting of a vehicle state estimator and integrated TRFC estimation was developed in [61], and the mean-square-error-weighted fusion-based UKFs were shown to provide high estimation accuracy of TRFC by mixing the estimation of the longitudinal and the lateral UKFs [62]. The work [63] presented the novel UKF to estimate TRFC and vehicle longitudinal and lateral velocities with standard vehicle dynamics control sensors. The experimental tests showed that the designed UKF clearly outperformed the EKF in terms of estimation accuracy and robustness.

The dual unscented Kalman filter (DUKF) based on the double track model and Dugoff tire was introduced to estimate vehicle sideslip angle and the key parameter of vehicle mass at CG [64]. DUKF, together with the double-track model and Pacejka tire model, was designed in order to simultaneously estimate the side slip angle, tire-road forces, and Pacejka tire parameters; then, the hybrid of the Levenberg–Marquardt and quasi Newton method was employed to identify the Pacejka tire coefficients [65].

*4) Cubature Kalman Filter(CKF)-based estimation*. The work [81] proposed a new nonlinear Kalman filter called the cubature Kalman filter (CKF) that solved the problem of "nonlinear function × gaussian density" in Bayesian filtering by using third-order spherical-radial cubature criterion, which can avoid the high-order Taylor truncation error in EKF and the instability of UKF caused from non-positive definite covariance in higher order non-linear systems. Several studies have tried to estimate vehicle states and parameters with the CKF technique [66,67,68,69,70,71]. The CKF method modeled from the double-track vehicle dynamics model and Dugoff tire was introduced in [67] to estimate vehicle sideslip angle and lateral tire forces by utilizing real-time measurements from standard sensors for distributed drive electric vehicles, and new adaptive CKF (ACKF) for estimating vehicle lateral and longitudinal velocities were found to present higher performance of anti-nonlinearity and noise when compared with the EKF and UKF methods [68]. In [69], the adaptive square-root CKF (ACKF)-based vehicle sideslip angle estimator was developed to adaptively adjust for the vehicle dynamics nonlinearity and model uncertainty using the integral estimation with the zero-point-reset method. To obtain vehicle states as well as unknown road condition [70], the dual cubature Kalman filter (DCKF) and joint cubature Kalman filter (JCKF) were designed to estimate vehicle lateral and longitudinal velocities and TRFC at each wheel. A novel IMM approach investigated in [71] was the hybrid estimator formed by fusing the square-root CKF and horizon Kalman filter with the square-root cubature method to estimate vehicle sideslip angle.

#### 2.2.2. Other Filter-Based Estimations

*1) Particle Filter (PF)-based estimation*. It is worth noting that the basic assumption of the aforementioned different types of Kalman filters is that the process noise and measurement noise of the system belongs to the Gaussian distribution. Whereas the real driving conditions and vehicle environments are complex, the Gaussian assumption-based Kalman filter is not always correct in practice. To address this issue, several other filters have been developed to estimate vehicle states. On the basis of the Monte Carlo and importance sampling assumptions, a new particle filter (PF) [72,73] with non-Gaussian distribution by using numerous particles was proposed for vehicle dynamic sideslip angle and tire lateral and longitudinal forces estimation, providing better precision when compared with EKF from experimental validations. To make up the shortage of sample impoverishment in PF, the unscented particle filter (UPF) was reported in [74] to estimate IWM vehicle states, such as vehicle sideslip angle and lateral tire force, in the high-order nonlinear vehicle dynamic system.

*2) Other filter-based estimations*. A moving horizon estimation (MHE) for vehicle sideslip angle has been introduced under corrupted measurement noise [75]. The online implementation problem of the MHE methodology in complex vehicle dynamics was also optimized using nonlinear finite impulse response (NFIR) filters [76]. The approach adopted in [77] was that of the state-dependent Riccati equation (SDRE)-based nonlinear filter on sideslip angle estimation to fully consider vehicle dynamic nonlinearities and measurement noise. In [78], the EKF and EHF filters under minimizing separate criteria were studied as estimators of vehicle sideslip angle and tire cornering stiffness. The performance of two estimators were compared according to the criterion of mean squared error and steady-state error. The work [79] proposed nonlinear constrained MHE to estimate mainly the vehicle position ***P_p_*** and vehicle sideslip angle for future autonomous vehicles; the delayed measurements from the global navigation satellite system (GNSS) and road boundary constraints can be directly incorporated into MHE, and real-world experiments show that the proposed MHE possesses improved estimation performance in comparison to the EKF.

### 2.3. Observer-Based Vehicle State Estimation

#### 2.3.1. Recursive Least Squares Method

The recursive least squares (RLS) method was applied to identify wheel slip corresponding to the peak of the TRFC in [82] and individual wheel TRFC estimation in [83], studied in order to provide real-time information for braking systems, traction control, and yaw stability control. RLS with forgetting factors were developed for implementing estimation of tire cornering stiffness and vehicle sideslip angle for IWM electric vehicles using onboard sensors [84,85], and a resonance frequency-based RLS was presented to estimate TRFC by considering the frequency response of the IWM drive system dynamics [86]. The linearized recursive least squares (LRLS) method in [87] identified the TRFC and tire cornering stiffness by the combined lateral and longitudinal tire model, making full use of frictional limits, and the proposed TRFC estimator with RLS [88] was based on measured 6-DOF vehicle body accelerations and accelerations at the tire.

**Table 2:** Methodologies, models, estimations and sensor configurations for observer-based vehicle state estimation.

Methodologies	Models	Estimations	Sensor Configurations	References
RLS	Longitudinal model + Burchhardt tire model	*μ*	*ω_ij_*, *a_x_*	[82]
RLS	Longitudinal model + Dugoff tire model	*μ*, *F_x_*	*V_x_*, *a_x_*, GPS	[83]
RLS	Single-track +Linear tire model	*β, C_α_*	*r_z_*, *δ_f_, a_x_*, *a_y_*,*ω_ij_*, *T_ij_*, *F_yij_*, MSU	[84]
RLS + NLO	Single-track + Linear tire model	*β, C_α_*	*r_z_*	[85]
RLS	Longitudinal model + Linear tire model	*μ*	*V_x_*, *T_m_*, *ω_ij_*	[86]
LRLS + KF	Double-track + Brush tire model	*β*, *F_x_*, *F_y_*, *F_z_*, *μ*, *C_α_*, *C_s_*	*r_z_*, *a_x_*, *a_y_*, *ω_ij_*	[87]
RLS	Double-track + Suspension model	*μ*	*a_x_*, *a_y_*, *a_z_*	[88]
LO	Single-track + Linear tire model	*β*	*r_z_*, *a_y_*	[89]
SOLEO	Longitudinal model + Burchhardt tire model	*μ*	*ω_ij_*, *T_b_*	[90]
HO/RO	Single-track + Linear tire model	*β*	*r_z_*	[91,92]
COO	Double-track + Suspension model	*C_α_*, *F_z_*	*a_y_*, *r_z_*, *p*	[93]
TFO	Longitudinal model + Pacejka tire model	*μ*, *F_x_*	*ω_ij_*, *V_x_*	[94]
FDO, RAO	Single-track + Brush tire model	*μ*	*F_y_*	[95,96]
SMO + RLS	Longitudinal model + Brush tire model	*μ*, *F_x_*	*a_x_*,*ω_ij_*, *T_m_*	[97]
SMO	Double-track + Roll + Dugoff tire model	*β,φ* , *F_x_*, *F_y_*, *F_z_*	*V_x_*, *V_y_*, *r_z_*, *a_x_*, *a_y_*, *p*	[98]
SMO	Rotational model of wheel + LuGre tire model	*μ*	*ω_ij_*	[99,100,101,102]
SMO	Rotational model of wheel + LuGre tire model	*μ*, *V_x_*	*ω_ij_*, *T_m_*	[103]
SOSMO	Rotational model of wheel + Pacejka tire model	*C_s_*	*ω_ij_*	[104]
SOSMO	Longitudinal model + LuGre model	*μ*	*ω_ij_*	[105]
VSSMO	Single-track + Linear tire model	*F_x_*, *F_y_*	*r_z_*, *a_x_*, *a_y_*	[106]
ROSMO	Double-track + UniTire tire model	*β*, *F_y_*, *F_z_*	*r_z_*,*ω_ij_*	[107]
HOSMO	Rotational model of wheel + LuGre tire model	*μ*	*ω_ij_*, *T_m_*	[108]
HOSMO	Double-track + Pacejka tire model	*β, F_x_*	*r_z_*,*a_x_*, *a_y_*	[109]
HOSMO	Single-track + Roll + Linear tire model	*F_z_*	*a_z_*	[110]
NLO	Double-track + Dugoff tire model	*V_x_*, *V_y_*	*r_z_*, *δ_f_, a_x_*,*a_y_*,*ω_ij_*	[111]
NLO	Double-track + Pacejka tire model	*V_x_*, *V_y_*	*r_z_*, *δ_f_, a_x_*, *a_y_*,*ω_ij_*	[112]
RNLO	Double-track + UniTire tire model	*V_x_*, *V_y_*	*r_z_*,*ω_ij_*, *δ_f_*	[113]
ANLO	Double-track + Parametrized friction model	*β*	*r_z_*, *δ_f_, a_x_*, *a_y_*, *ω_ij_*	[114]
HNLO	Single-track + Pacejka tire model	*β*	*r_z_*, *δ_f_, V_x_*, *a_y_*	[115]
NLO	Single-track + Other nolinear tire model	*β*	*r_z_*, *δ_f_, V_x_*,*a_y_*	[116,117,118,119]
UIO	Roll model	*φ*,*θ*	*V_x_*, *V_y_*, *r_z_*, *z_ij_*,*p*	[120]
NLO	Single-track + Linear tire model	*β*	*V_g_*, *a_y_*	[121]
SNLO	Double-track + Dugoff tire model	*V_x_*, *V_y_*, *μ*	*r_z_*, *a_x_*,*a_y_*,*ω_ij_*	[122,123]
NLO	Single-track + Other nolinear tire model	*V_x_*, *V_y_*	*ω_ij_*, *T_m_*,*a_x_*,*a_y_*	[124]
NLO	Rotational model of wheel + LuGre tire model	*μ*, *F_x_*	*ω_ij_*, *T_m_*	[125]
NLO	Single-track + Brush tire model	*μ, C_α_*	*ω_ij_*, *T_m_*	[126]
NLO	Brush tire model	*μ*	*a_y_*, WPS	[127,128]

#### 2.3.2. Linear Observer

The work [89] studied Luenberger observer for estimating vehicle sideslip angle and lateral states; its estimation accuracy was experimentally tested using a laboratory car equipped with the non-contact optical sensor "Correvit S-400". A second order linear extended state observer (SOLEO) from braking dynamic was proposed in [90] to estimate TRFC, utilizing braking torque of front and rear wheels as excitation. The study in [91] designed an optimal sideslip angle observer via finite-frequency H_∞_ approach (HO) considering the frequency of the front-wheel steering angle measured from an IWM electric vehicle-equipped navigation system, wherein a robust sideslip angle observer (RO) established from a singular vehicle model was developed by applying the uncertain singular method for model uncertainty [92]. The controller output observer (COO) in [93] that derived the observer model with inputs was designed to estimate vehicle tire cornering and normal forces. A tire traction forces observer (TFO) that was robust with respect to variations of vehicle parameter uncertainties and road conditions was presented for guaranteeing desired traction forces in vehicle control systems [94]. Four different TRFC observers (FDO) with different lateral and longitudinal excitation conditions were integrated to improve robustness of observers by expanding the working range [95], whereas the research in [96] found that vehicle tire slip angle was highly coupled with TRFC, a robust adaptive observer (RAO) was decoupled for vehicle tire slip angle estimation coupled with TRFC, and its robustness and convergence can be guaranteed using Lyapunov theorem.

#### 2.3.3. Sliding Mode Observer

General sliding mode observers (SMO) [97,98,99,100,101,102,103], second order sliding mode observer (SOSMO) [104,105], variable gain sliding mode control observer (VSSMO) [106], reduced-order sliding mode observer (ROSMO) [107], and higher-order sliding mode observer (HOSMO) [108,109,110] have been widely investigated for vehicle dynamic model-based state and parameter estimation, and experiments have confirmed that the proposed SMO can provide higher estimation accuracy when compared with EKF [100,109]. General SMO using the so-called equivalent control technique in observer is developed to estimate longitudinal forces [97], full tire forces and roll angle [98], and TRFC [99,100,101,102]. The SMO of combined vehicle velocity and TRFC estimation was designed by exploiting the low frequency component of the observer error dynamics [103]. The SOSMO using robust observer of supertwisting in nominal model was developed to identify tire stiffness and effective wheel [104], as well as TRFC [105]. VSSMO for estimating tire–road forces as unknown inputs was presented through on-board low-cost sensors from CAN-bus in heavy duty vehicles [106]. To reduce the implement load and chattering in vehicle lateral dynamics estimation, ROSMO in [107] was developed for vehicle slip angle and tire-road force estimation considering vehicle load transfers. On the basis of quarter vehicle dynamics integrated from LuGre friction tire, HOSMO in [108] was designed to observe TRFC as unknown input estimation, which was established with convergence of the estimation error under Lipschitz conditions and solved in terms of the supertwisting algorithm. The HOSMO presented in [109] was used to estimate vehicle sideslip angle and longitudinal force of IWM electric vehicles, and it was reconstructed by model decoupling and the electric driving wheel model, whereas vertical forces that can calculate the load transfer ratio (LTR) of heavy-vehicles for rollover risk prediction was observed by the HOSMO, and its performance was validated experimentally within many scenarios [110].

#### 2.3.4. Nonlinear Observer

In order to directly deal with nonlinear problems in vehicle dynamics state estimation, concerns about nonlinear observer (NLO) tend to increase, and sufficient conditions for this type of observer are derived by making use of different stability theories [111,112,113,114,115,116,117,118,119,120,121,122,123,124,125,126,127,128]. Experimental tests have shown that the estimated performance of the NLO is generally better than that of the EKF [111,113,115]. On the basis of the input-to-state stability (ISS) theory [111,112,113], nonlinear observer of longitudinal and lateral velocities have been proposed. NLO that uses the error between measured and estimated lateral and longitudinal accelerations as the feedback term was developed in [111,112], and the work [113] constructed the reduced-order NLO (RNLO) wherein yaw rate was selected as a function of vehicle velocities, and the dynamics system error, including mass and CG variation, was considered as additive disturbance inputs of the ISS system. In [114], the adaptive vehicle sideslip angle NLO (ANLO) that is uniformly globally asymptotically stable and locally exponentially stable was designed using Matrosov theorem under some technical assumptions and a parametrized road–tire friction model, and high-gain NLO (HNLO) of vehicle sideslip angle for adapting tire–road friction was presented by input–output linearization [115]. With the help of mean value (MV) theorem [116,117,118,119], vehicle sideslip angle NLO design described with uniformly bounded error vehicle dynamics was asymptotically stable by simplifying the nonlinear tire model [116], whereas the vehicle sideslip angle NLO in [117] was treated as a differentiable nonlinear system with a globally bounded Jacobian through modifying the MV theorem. In [120], the road bank was estimated with an unknown input observer (UIO) that was based on the roll dynamics implemented via dynamic fault thresholds algorithm. Using the singular values of this observability matrix, tire cornering stiffness was identified from vehicle sideslip angle NLO [121]. The research [122,123] introduced novel-switched NLO (SNLO) to estimate vehicle sideslip angle and TRFC simultaneously using the simplified Pacejka tire model or rational tire model through Lyapunov function. With extended information and architecture of four-in-wheel-motor-drive electric vehicles [124,125,126,127], the concept of effective inertia considering in-wheel-motor information was constructed to estimate the vehicle state of an electric vehicle [124]. On the other hand, a slip-based NLO with known motor torque in [125] was designed to estimate the tire longitudinal force and TRFC with Lyapunov stability theory, whereas tire cornering stiffness and TRFC estimation that was based on longitudinal force difference between the left and right wheels of in-wheel-motor-drive electric vehicles was proposed via algebraic techniques [126]. There are also some TRFC estimation methods that utilize tire lateral deflection measured from additional sensors embedded inside the wireless piezoelectric sensor (WPS) through the contact patch, for example, in [127,128].

## 3. Data-Driven-Based Vehicle Estimation

**Figure 6:**
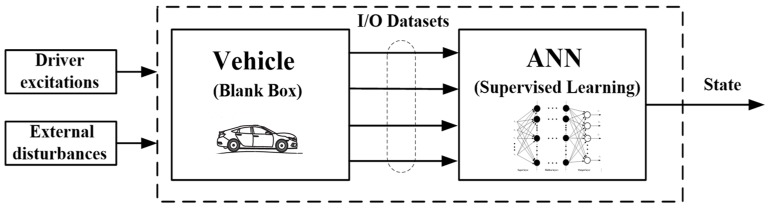
The schematic of artificial neural network (ANN) estimation process.

Data-driven estimation approaches are designed and constructed to estimate vehicle state and parameters on the basis of data that consists of historical and online I/O datasets in the vehicle dynamics system, and they gain inherited knowledge in capturing vehicle dynamic characteristics through processing these datasets [129,130,131]. In contrast to the aforementioned vehicle model-based estimations, the data-driven methods do not depend on the reference vehicle models, and they have been proven to possess the ability to avoid issues in vehicle dynamics estimation [129,130,131]. Among advanced data-driven approaches, artificial neural network (ANN)-based artificial intelligence (AI) is the most popular data-driven method for estimating vehicle state [129,130,131,132], the schematic of artificial neural network (ANN) estimation process is shown in Figure 6, which shows promising perspectives in various estimation applications such as energy estimation of ground vehicles [133], underwater vehicles [134], hypersonic vehicles [135], and unmanned aerial vehicles [136]. The main data-driven-based vehicle state estimations are summarized in Table 3.

In [137], the adaptive neuro-fuzzy inference system (ANFIS) with input delay technique was developed to estimate vehicle velocity and position through the fusion of datasets from the GPS and inertial navigation system (INS); experimental results have demonstrated that ANFIS can provide improved estimation accuracy when compared with the EKF method. The NNs have also been employed to estimate vehicle states by fusing multi-sensors [138,139,140,159]. The integration of GPS/INS through NNs considered in [139] was done to process the GPS signal in case of INS signal loss so that it can obtain accurate position and data, whereas the neural network-based MMF in [140] was adopted in order to obtain accurate and reliable position estimation of autonomous vehicles by combining GPS and on-board sensors. The deep learning (DL)-based GNSS network was also structured to improve the GNSS absolute positioning accuracy of automatic navigation of vehicles by combining various sensors [141].

Different ANN methods have been designed for estimating vehicle sideslip angle [142,143,144,145,146]. The works [142,143] presented ANFIS methodology to estimate pseudo-sideslip angle through measured parameters from steering wheel and inertial sensors, and experimental results showed that ANFIS can learn behaviors of vehicle sideslip angle reliably without difficulty, supplying more reliable estimations of sideslip angle than Kalman filters. Deneral regression neural network (GRNN) estimator that derived from the RBF neural network using enhanced output of radial basis layer in [144] has been proposed; its homogeneous design is used to optimize the training samples of the driver–vehicle closed-loop system, wherein vehicle experiments verified the validity of GRNN where test amplitudes for mean of error are within 10%. On the basis of the feed forward and dynamic NN, the improved Elman neural network (IEMM) in [145] was introduced by adding the context nodes; it can be more sensitive to the historical data under self-linking between the hidden nodes of input and output in NN; thereby, IEMM possesses increased processing capability for dynamic information for vehicle sideslip angle estimation. The research in [146] addressed influences of vehicle velocity variations and various tire–road adherence conditions on NN performance of vehicle sideslip angle estimation; it is designed so that the NNs with 1 hidden layer of 10 neurons and a single output neuron where vehicle velocity, yaw rate, lateral acceleration, and steering angle are used as inputs, and the training sets of NNs are characterized by different clockwise and anti-clockwise maneuvers under high and low friction roads to increase its reliability; then, performance and robustness of the NNs are subsequently studied with experimental datasets. In [147], a deep learning network-based sideslip angle observer was introduced, and professional road tests show that observer can accurately estimate the sideslip angle against various handling conditions.

To predict the lateral load transfer and roll stability vector in roll stability control system, the NN-based roll angle estimations in [148,149,150] were taken into account using IoT low-cost devices and IMU where longitudinal and lateral accelerometer, yaw rate, and roll rate can be easily measured as training set, and the NN embedded in IoT low-cost devices can handle real-time constraints. Herein, the experimental result verified that the NN can obtain improved accuracy during the estimating process of vehicle roll angle with respect to KF [150]. 

Aiming the estimation of tire–road forces and interaction between the tire and road, the feedforward NN (FNN) in [151] was used to estimate the lateral tire forces of nonlinear tire behavior without the tire–road friction model, whereas the multilayered perceptron NN (MPNN) with backpropagation algorithm estimated traction forces in [160] by using experimental datasets. The neural network-based tire–road friction force estimators emphasized in [152] were to improve estimation sensitivity of friction forces for friction adaptiveness. A novel progressive NN (PNN) was trained to determine vertical suspension force factors on the basis of the dynamic response of the tire and road interaction [153]. In [154], a neural network with the gradient descent method (NN-GD) was developed to represent lateral forces of tire dynamics, and the NN with 13 hidden neurons used a range of tire pressures, slip angles, normal forces, and inclination angles as trained inputs with 100,000 tire data points under combined slip conditions, which can generate lateral forces similar to the empirical models. By considering bounds or constraints of the input data, such as slip angle and other input data, ANNs and the Nelder–Mead-based method in [155] were compared and studied to estimate coefficients of the magic formula tire model. The deep learning method composed of the convolutional neural network and recurrent neural network was proposed to enhance the reliability of estimating the TRFC under different driving situations [157].

The ANN approach in [161] has also been reported as a solution to estimate the road profile in order to replace laser sensors or response-type road roughness measurements in vehicle dynamic systems, and the multi-input and multi-output feed forward wavelet neural network (WNN) wherein wavelet basis functions are utilized as activate function in hidden layer and back propagation (BP) algorithm applied in the training process was further developed for road profile estimation [162]. To predict the limit and margin of vehicle in road safety, this work [163] was done to develop NN methodology to identify the tire–road maximal friction coefficient by estimating forces and aligning the torque of tires. An ANN was introduced to detect the road condition through estimating the optimal slip [157]. Multilayer perceptron neural network (MPNN) was presented to obtain the road friction coefficient, which was trained using known parameters from the magic formula tire model on the basis of other estimated states, such as vehicle lateral and longitudinal velocities and lateral and longitudinal tire forces [158].

Aside from this, NNs have been extended to other vehicle dynamic estimations [164,165,166]. For instance, trained NNs in [164] were employed to identify three body-dominated motion-modes consisting of roll, bounce, and pitch motion-modes, which can avoid the requirements of measurement for full vehicle states and road inputs when compared with the motion-mode energy-based identification method. ANNs with multi-layer feedforward network (MLFN) and GRNN have been introduced to improve evaluation stability of vehicle handling that is always difficult to be assessed using determined impact factors [165]. The multilayer perceptron NN with a single hidden layer presented in [166] was used to estimate driver activity regardless of the vehicle and tire models.

## 4. Conclusions

In this paper, a comprehensive survey of developments and latest advances for vehicle dynamics state estimation is presented. Different aspects of estimation strategies and methodologies in the most recent literature are reviewed and classified into two main categories consisting of the model-based estimation approach and data-driven-based estimation approach. The development of the most popular estimation models, techniques, and approaches, which are widely used to estimate vehicle dynamics states, are discussed and analyzed. These estimation approaches and methodologies discussed in this paper will help researchers to obtain an overview for further research in this field. Although numerous achievements for vehicle dynamic state estimation have been provided in the literature, there are still some open research questions that should be further considered in future works. Two topics are highlighted and discussed here.

*1) The range of estimation can be extended towards connected and automated driving vehicles*. Emerging connected and automated driving vehicles have appeared as promising vehicle architectures based on several advantages in terms of good safety, high energy efficiency, and road traffic efficiency [13,14,15]. The connected autonomous vehicles require understanding of vehicle-to-human interaction and human driver behavior for human–machine sharing control; thus, it is essential to identify driver driving behavior (e.g., driver distraction, fatigue) and driver intention towards increasing automated driving levels of vehicle. Because driver driving behavior and driver intention can be influenced by a large number of factors, such as inter-responses of the drive and the external stressing conditions, the process of accurately recognizing driver driving behavior and driver intention on the basis of vehicle operating states is a promising challenge. ANN-based machine learning may be a good option, as it possesses the knowledge processing and learning capability of the human brain that can adapt to identify complex driver driving behavior and driver intention [167,168].

*2) The nonlinear challenge of vehicle system dynamic state estimation*. Vehicle dynamics system is an inherently nonlinear system—vehicle and tire dynamics present high nonlinearities especially when a vehicle undergoes high accelerations under extreme handling conditions. How to deal with nonlinear estimation problems in vehicle dynamics is still an open research challenge. There are two typical types of nonlinear estimation techniques found in the previously discussed literature—one that uses linearization methods such as Taylor truncation in EKF to linearly approximate the nonlinear vehicle dynamics estimation system, and the other that directly designs a nonlinear observer to deal with a nonlinear problem of the vehicle dynamics estimation system. It is worth noting that despite the observability of the linearization-based estimations being easily demonstrated using the linear system stability theory, the estimation accuracy of the method for vehicle state needs to be further improved. Perhaps the nonlinear observer provides an acceptable estimation accuracy, whereas the global observability of a nonlinear observer is generally difficult, and local observability for this type of observer also faces some challenges. With the rapid development of nonlinear estimation theory, input-to-state stability theory [111,112,113], Matrosov theorem [114], and other stability theories [116,117,118,119] this problem is gradually being solved. Moreover, the data-driven-based estimation possesses the potential to enhance vehicle dynamics state estimation, and the additional attention on developments and applications of data-driven-based estimation should be paid to further improve performance for vehicle dynamics estimation systems in the future.

## Figures and Tables

**Table 1:** Methodologies, models, estimations and sensor configurations for filter-based vehicle state estimation.

Methodologies	Models	Estimations	Sensor Configurations	References
KF	Single-track + Linear tire model	*V_y_*	*r_z_*, *δ_f_*	[21]
KF + RLS	Single-track + Roll + Linear tire model	*β*, *φ*	*r_z_*, *δ_f_, a_x_*, *a_y_*, *ω_ij_*, *T_ij_*, *F_yij_*, MSU	[22]
KF	Single-track + Linear tire model	*β*	*r_z_, ψ,* GPS	[23,24,25]
DKF	Single-track + Linear tire model	*C_α_*	*V_x_*, *V_y_*, *ψ,* GPS	[26]
RWKF	Double-track + Linear tire model	*F_x_*, *F_y_*	*r_z_*, *a_x_*, *a_y_*, *ω_ij_*	[27]
EKF	Single-track + Pacejka tire model	*β*	*r_z_*, *a_y_*	[28]
EKF	Single-track + Linear tire model	*β, C_α_*	*r_z_*, *F_yij_*, MSU	[29]
EKF	Single-track + Pacejka tire model	*β*	*T_δf_*, *r_z_*, *a_x_*, *a_y_*	[30]
EKF + SMC	Single-track+Roll+Dugoff tire model	*β*, *φ*	*V_x_, r_z_*, *a_x_*, *a_y_*	[31]
EKF	Single-track + Pacejka tire model	*β*	*r_z_*, *a_y_*	[32]
EKF	Double-track + Roll + Pacejka tire model	*V_x_*, *V_y_*, *φ*, *F_y_*, *DBE*	*r_z_*, *a_x_*, *a_y_*, *p*	[33,34]
EKF	Double-track + Dugoff tire model	*β*, *F_y_*	*r_z_*, *δ_f_*, *a_x_*, *a_y_*, *ω_ij_*	[35]
VSEKF	Double-track + Dugoff tire model	*β*	*r_z_*, *δ_f_, a_y_*	[36]
EKF	Double-track + Roll + Dugoff tire model	*β*, *F_y_*, *F_z_*	*r_z_*, *a_x_*, *a_y_*, *p*	[37]
EKF	Double-track + Pacejka tire model	*V_x_*, *V_y_*	*r_z_*, *δ_f_, a_x_*, *a_y_*, *ω_ij_*	[38]
EKF + MME	Double-track + Pacejka tire model	*V_x_*, *V_y_*, *F_x_*, *F_y_*	*r_z_*, *δ_f_, a_y_*	[39]
EKF	Single-track + Burchhardt tire model	*β*, *F_x_*, *F_y_*, *μ*	*r_z_*, *δ_f_, a_x_*, *a_y_*, *ω_ij_*	[40]
EKF	Single-track + Pacejka tire model	*β*	*r_z_*, *a_y_*	[41]
EKF	Longitudinal model + Pacejka tire model	*μ*	*a_y_*, *ω_ij_*	[42,43]
DEKF	Double-track + Roll + Pacejka tire model	*μ*, *F_x_*, *F_y_*, *F_z_*	*r_z_*, *a_x_*, *a_y_*, *p*	[44]
EKF	Single-track + Roll + Linear tire model	*β*, *φ*	*r_z_*, *δ_f_, a_x_*, *a_y_*, *ψ,* GPS	[45,46]
EKF	Single-track + Brush tire model	*β*, *μ*	*V_x_*, *V_y_*, *ψ*, *r_z_*, *a_x_*, *a_y_*, *p*, GPS	[47]
EKF	Single-track + Pacejka tire model	*V_x_*, *β*, *θ*, *C_α_,μ*	*r_z_*, *δ_f_, a_x_*, *a_y_*, *ω_ij_*	[48]
DEKF	Double-track + Pacejka tire model	*β*, *m*, *I_zz_*	*r_z_*, *a_y_*, *V_x_*	[49]
DEKF	Single-track + Roll + Linear tire model	*β, φ*, *C_α_*, *I_zz_*	*r_z_*, *δ_f_, a_x_*, *a_y_*, *p*	[50]
DEKF	Double-track +Dugoff tire model	*β*, *μ*	*r_z_*, *a_x_*, *a_y_*	[51]
IMM-EKF	Single-track + Other nolinear tire model	*β*, *μ*	*r_z_*, *a_y_*	[52]
IMM-UKF	Double-track + Roll + Dugoff tire model	*β*,*φ*	*r_z_*, *a_x_*, *a_y_*, *p, ω_ij_*	[53]
IMM-EKF	Single-track + Other nolinear tire model	*β*, *F_x_*, *F_y_*	*r_z_*, *δ_f_*, *V_x_*, *a_y_*	[54]
UKF	Double - track+ UniTire tire model	*V_x_*, *V_y_*	*r_z_*, *a_x_*, *a_y_*, *ω_ij_*	[55]
UKF	Single-track + Linear tire model	*β*	*a_x_*, *a_y_*	[56]
AUKF	Double-track + Pacejka tire model	*β*	*a_y_*, *r_z_*	[57,58]
CUKF	Single-track + Random Walk model	*β*, *F_y_*	*r_z_*, *a_y_*	[59]
UKF/EKF	Double-track + Dugoff tire model	*β*, *F_y_*	*r_z_*, *δ_f_, a_x_*, *a_y_*, *ω_ij_*	[60]
UKF	Double-track + Dugoff tire model	*μ*	*a_x_*, *a_y_*	[61,62]
UKF	Double-track + Pacejka tire model	*V_x_*, *V_y_*,*μ*	*r_z_*, *a_y_*, *ω_ij_*	[63]
DUKF	Double-track + Dugoff tire model	*β*, *m*	*r_z_*, *a_y_*, *V_y_*	[64]
DUKF	Double-track + Roll + Pacejka tire model	*β*, *φ*, *F_y_*, *F_z_*, *DBE*	*r_z_*, *a_x_*, *a_y_*, *p, ω_ij_*	[65]
CKF	Single-track + Linear tire model	*β*	*δ_f_, a_y_*	[66]
CKF	Double-track + Roll + Dugoff tire model	*β*,*φ*, *F_x_*, *F_y_*	*r_z_*, *a_x_*, *a_y_*, *p, ω_ij_*	[67]
ACKF	Double-track + Pacejka tire model	*V_x_*, *V_y_*	*r_z_*, *δ_f_, a_x_*, *a_y_*, *ω_ij_*	[68,69]
JCKF, DCKF	Double-track + Pacejka tire model	*V_x_*, *V_y_*,*μ*	*r_z_*, *a_x_*, *a_y_*	[70]
IMM+CKF	Double-track + Pacejka tire model	*V_x_*, *V_y_*	*r_z_*, *a_x_*, *a_y_*	[71]
PF	Double-track + Dugoff tire model	*β*, *F_x_*, *F_y_*	*r_z_*, *a_x_*, *a_y_*	[72,73]
UPF	Double-track + Pacejka tire model	*β*, *F_y_*	*r_z_*, *a_x_*, *a_y_*	[74]
MHE	Single-track + Pacejka tire model	*β*	*r_z_*	[75,76]
SDRE + EKF	Single-track + Random Walk model	*β*	*r_z_*, *a_y_*	[77]
EHF	Single-track + Linear tire model	*β, C_α_*	*r_z_, ψ,* GPS	[78]
MHE	Single-track + Pacejka tire model	*β*, *P_p_*	*r_z_*, *P_c_*, GNSS	[79]

**Table 3:** Methodologies, estimations and trained inputs for data-driven-based vehicle state estimation.

Methodologies	Estimations	Trained Inputs	References
ANFIS	*V_x_*, *P_p_*	*P_c_*, GPS, IMU	[137]
NN	*φ*	*r_z_*, *a_x_*, *a_y_*, *p*	[138]
NN	*P_p_*	*P_c_*, GPS, IMU	[139,140]
DL	*P_p_*	*P_c_*, GPS, IMU	[141]
ANFIS	*β*	*r_z_*, *δ_f_, a_x_*, *a_y_*	[142,143]
GRNN	*β*	*r_z_*, *a_y_*	[144]
IEMM	*β*	*r_z_*, *δ_f_, a_y_*	[145]
NN	*β*	*r_z_*, *δ_f_, V_x_*, *a_y_*	[146]
DL	*β*	*r_z_*, *δ_f_, a_x_*, *a_y_*,*ω_ij_*	[147]
NN	*φ*	*r_z_*, *a_x_*, *a_y_*, *p*	[148,149,150]
FNN	*F_y_*	*a_x_*,*α*	[151]
NN	*F_x_*	*V_x_*, *a_x_*,*ω_ij_*	[152]
PNN	*F_z_*	*a_z_*, *z_m_*	[153]
NN-GD	*F_y_*	*α, P_t_, F_z_*	[154]
NN	*B, D, E*	*F_x_*, *F_y_*,*α*, *s*	[155]
DL	*μ*	*r_z_*, *δ_f_, a_x_*, *a_y_*, *a_z_α*,*s*,*ω_ij_*	[156]
NN	*M_x_*	*F_x_*, *F_y_*, *F_z_, δ_f_*	[157]
MPNN	*μ*	*F_x_*, *F_y_*, *F_z_,α*,*s*	[158]

## References

[1] Goodarzi A., Esmailzadeh E. (2007). Design of a VDC system for all-wheel independent drive vehicles. IEEE/ASME Trans. Mechatron..

[2] Jin X., Yu Z., Yin G., Wang J. (2017). Improving vehicle handling stability based on combined AFS and DYC system via robust Takagi-Sugeno fuzzy control. IEEE Trans. Intell. Transp. Syst..

[3] Poussot-Vassal C., Sename O., Dugard L., Savaresi S.M. (2011). Vehicle dynamic stability improvements through gain-scheduled steering and braking control. Veh. Syst. Dyn..

[4] Zhang H., Zhang X., Wang J. (2014). Robust gain-scheduling energy-to-peak control of vehicle lateral dynamics stabilisation. Veh. Syst. Dyn..

[5] Perić S.L., Antić D.S., Milovanović M.B., Mitić D.B., Milojković M.T., Nikolić S.S. (2015). Quasi-sliding mode control with orthogonal endocrine neural network-based estimator applied in anti-lock braking system. IEEE/ASME Trans. Mechatron..

[6] Wei Z., Guo X. (2014). An ABS control strategy for commercial vehicle. IEEE/ASME Trans. Mechatron..

[7] Tan Q., Shi L., Katupitiya J. (2019). A novel control approach for path tracking of a force-controlled two-wheel-steer four-wheel-drive vehicle. Proc. Inst. Mech. Eng. D J. Autom. Eng..

[8] Shu P., Sagara S., Wang Q., Oya M. (2017). Improved adaptive lane-keeping control for four-wheel steering vehicles without lateral velocity measurements. Int. J. Robust Nonlinear Control.

[9] Jin X., Yin G., Bian C., Chen J., Li P., Chen N. (2016). Gain-scheduled vehicle handling stability control via integration of active front steering and suspension systems. ASME Trans. J. Dyn. Syst. Meas. Control.

[10] Li H., Liu H., Gao H., Shi P. (2011). Reliable fuzzy control for active suspension systems with actuator delay and fault. IEEE Trans. Fuzzy Syst..

[11] Weißmann A., Görges D., Lin X. (2018). Energy-optimal adaptive cruise control combining model predictive control and dynamic programming. Control Eng. Pr..

[12] Lian Y., Zhao Y., Hu L., Tian Y. (2015). Longitudinal collision avoidance control of electric vehicles based on a new safety distance model and constrained-regenerative-braking-strength-continuity braking force distribution strategy. IEEE Trans. Veh. Technol..

[13] Zheng Y., Li S.E., Wang J., Cao D., Li K. (2015). Stability and scalability of homogeneous vehicular platoon: Study on the influence of information flow topologies. IEEE Trans. Intell. Transp. Syst..

[14] Hu C., Wang Z., Taghavifar H., Na J., Qin Y., Guo J., Wei C. (2019). MME-EKF-Based Path-Tracking Control of Autonomous Vehicles Considering Input Saturation. IEEE Trans. Veh. Technol..

[15] Guanetti J., Kim Y., Borrelli F. (2018). Control of connected and automated vehicles: State of the art and future challenges. Annu. Rev. Control..

[16] Hac A., Simpson M.D. (2000). Estimation of vehicle side slip angle and yaw rate. SAE Techn. Pap..

[17] Piyabongkarn D., Rajamani R., Grogg J.A., Lew J.Y. (2009). Development and experimental evaluation of a slip angle estimator for vehicle stability control. IEEE Trans. Control Syst. Technol..

[18] Lee H. (2006). Reliability indexed sensor fusion and its application to vehicle velocity estimation. ASME Trans. J. Dyn. Syst. Meas. Control.

[19] Cheli F., Sabbioni E., Pesce M., Melzi S. (2007). A methodology for vehicle sideslip angle identification: Comparison with experimental data. Veh. Syst. Dyn..

[20] Chen B.C., Hsieh F.C. (2008). Sideslip angle estimation using extended Kalman filter. Veh. Syst. Dyn..

[21] Venhovens P.J.T., Naab K. (1999). Vehicle dynamics estimation using Kalman filters. Veh. Syst. Dyn..

[22] Nam K., Oh S., Fujimoto H., Hori Y. (2012). Estimation of sideslip and roll angles of electric vehicles using lateral tire force sensors through RLS and Kalman filter approaches. IEEE Trans. Ind. Electron..

[23] Anderson R., Bevly D.M. (2010). Using GPS with a model-based estimator to estimate critical vehicle states. Veh. Syst. Dyn..

[24] Nguyen B.M., Wang Y., Fujimoto H., Hori Y. (2013). Lateral stability control of electric vehicle based on disturbance accommodating kalman filter using the integration of single antenna GPS receiver and yaw rate sensor. J. Electron. Eng. Technol..

[25] Ryu J., Gerdes J.C. (2004). Integrating inertial sensors with global positioning system (GPS) for vehicle dynamics control. ASME Trans. J. Dyn. Syst. Meas. Control..

[26] Lee S., Nakano K., Ohori M. (2015). On-board identification of tyre cornering stiffness using dual Kalman filter and GPS. Veh. Syst. Dyn..

[27] Cho W., Yoon J., Yim S., Koo B., Yi K. (2009). Estimation of tire forces for application to vehicle stability control. IEEE Trans. Veh. Technol..

[28] Gadola M., Chindamo D., Romano M., Padula F. (2014). Development and validation of a Kalman filter-based model for vehicle slip angle estimation. Veh. Syst. Dyn..

[29] Nam K., Fujimoto H., Hori Y. (2012). Lateral stability control of in-wheel-motor-driven electric vehicles based on sideslip angle estimation using lateral tire force sensors. IEEE Trans. Veh. Technol..

[30] Ma B., Liu Y., Gao Y., Yang Y., Ji X., Bo Y. (2018). Estimation of vehicle sideslip angle based on steering torque. Int. J. Adv. Manuf. Technol..

[31] Han S., Huh K. (2011). Monitoring system design for lateral vehicle motion. IEEE Trans. Veh. Technol..

[32] Li X., Song X., Chan C. (2014). Reliable vehicle sideslip angle fusion estimation using low-cost sensors. Measurement.

[33] Kim J. (2010). Effect of vehicle model on the estimation of lateral vehicle dynamics. Int. J. Autom. Technol..

[34] Kim J. (2009). Identification of lateral tyre force dynamics using an extended Kalman filter from experimental road test data. Control Eng. Pr..

[35] Dakhlallah J., Glaser S., Mammar S., Sebsadji Y. Tire-road forces estimation using extended Kalman filter and sideslip angle evaluation. Proceedings of the American control conference.

[36] Li L., Song J., Li H., Zhang X. (2011). A variable structure adaptive extended Kalman filter for vehicle slip angle estimation. Int. J. Veh. Des..

[37] Doumiati M., Victorino A., Lechner D., Baffet G., Charara A. (2010). Observers for vehicle tyre/road forces estimation: Experimental validation. Veh. Syst. Dyn..

[38] Guo H., Chen H., Xu F., Wang F., Lu G. (2012). Implementation of EKF for vehicle velocities estimation on FPGA. IEEE Trans. Ind. Electron..

[39] Liu W., He H., Sun F. (2016). Vehicle state estimation based on Minimum Model Error criterion combining with Extended Kalman Filter. J. Frankl. Inst..

[40] Baffet G., Charara A., Dherbomez G. (2007). An observer of tire-road forces and friction for active security vehicle systems. IEEE/ASME Trans. Mechatron..

[41] Hodgson G., Best M.C. (2006). A parameter identifying a Kalman filter observer for vehicle handling dynamics. Proc. Inst. Mech. Eng. D J. Autom. Eng..

[42] Li B., Du H., Li W. (2014). Comparative study of vehicle tyre–road friction coefficient estimation with a novel cost-effective method. Veh. Syst. Dyn..

[43] Enisz K., Szalay I., Kohlrusz G., Fodor D. (2015). Tyre-road friction coefficient estimation based on the discrete-time extended Kalman filter. Proc. Inst. Mech. Eng. D J. Autom. Eng..

[44] Qi Z., Taheri S., Wang B., Yu H. (2015). Estimation of the tyre–road maximum friction coefficient and slip slope based on a novel tyre model. Veh. Syst. Dyn..

[45] Huang J., Tan H.S. (2006). A low-order DGPS-based vehicle positioning system under urban environment. IEEE/ASME Trans. Mechatron..

[46] Li X., Chan C.Y., Wang Y. (2015). A reliable fusion methodology for simultaneous estimation of vehicle sideslip and yaw angles. IEEE Trans. Veh. Technol..

[47] Yoon J.H., Li S.E., Ahn C. (2016). Estimation of vehicle sideslip angle and tire-road friction coefficient based on magnetometer with GPS. Int. J. Autom. Technol..

[48] Bechtoff J., Isermann R. (2016). Cornering stiffness and sideslip angle estimation for integrated vehicle dynamics control. Ifac-Pap..

[49] Wenzel T.A., Burnham K.J., Blundell M.V., Williams R.A. (2006). Dual extended Kalman filter for vehicle state and parameter estimation. Veh. Syst. Dyn..

[50] Cheng C., Cebon D. (2011). Parameter and state estimation for articulated heavy vehicles. Veh. Syst. Dyn..

[51] Zong C., Hu D., Zheng H. (2013). Dual extended Kalman filter for combined estimation of vehicle state and road friction. Chin. J. Mech. Eng..

[52] Tsunashima H., Murakami M., Miyataa J. (2006). Vehicle and road state estimation using interacting multiple model approach. Veh. Syst. Dyn..

[53] Jin X., Yin G. (2015). Estimation of lateral tire-road forces and sideslip angle for electric vehicles using interacting multiple model filter approach. J. Frankl. Inst..

[54] Jung H., Choi S.B. (2017). Real-time individual tire force estimation for an all-wheel drive vehicle. IEEE Trans. Veh. Technol..

[55] Zhao Z., Chen H., Yang J., Wu X., Yu Z. (2015). Estimation of the vehicle speed in the driving mode for a hybrid electric car based on an unscented Kalman filter. Proc. Inst. Mech. Eng. D J. Autom. Eng..

[56] Wang Y., Kang F., Wang T., Ren H. (2018). A robust control method for lateral stability control of in-wheel motored electric vehicle based on sideslip angle observer. Shock. Vibrat..

[57] Chen J., Song J., Li L., Jia G., Ran X., Yang C. (2016). UKF-based adaptive variable structure observer for vehicle sideslip with dynamic correction. Iet Control Theory Appl..

[58] Wang Z., Qin Y., Gu L., Dong M. (2017). Vehicle system state estimation based on adaptive unscented Kalman filtering combing with road classification. IEEE Access.

[59] Strano S., Terzo M. (2018). Constrained nonlinear filter for vehicle sideslip angle estimation with no a priori knowledge of tyre characteristics. Control Eng. Pr..

[60] Doumiati M., Victorino A.C., Charara A., Lechner D. (2010). Onboard real-time estimation of vehicle lateral tire-road forces and sideslip angle. IEEE/ASME Trans. Mechatron..

[61] Ren H., Chen S., Shim T., Wu Z. (2014). Effective assessment of tyre-road friction coefficient using a hybrid estimator. Veh. Syst. Dyn..

[62] Chen L., Bian M., Luo Y., Li K. (2016). Real-time identification of the tyre-road friction coefficient using an unscented Kalman filter and mean-square-error-weighted fusion. Proc. Inst. Mech. Eng. D J. Autom. Eng..

[63] Antonov S., Fehn A., Kugi A. (2011). Unscented Kalman filter for vehicle state estimation. Veh. Syst. Dyn..

[64] Cheng Q., Correa-Victorino A., Charara A. A new nonlinear observer of sideslip angle with unknown vehicle parameter using the dual unscented Kalman filter. Proceedings of the 15th International IEEE Conference on Intelligent Transportation Systems.

[65] Davoodabadi I., Ramezani A.A., Mahmoodi M.-K., Ahmadizadeh P. (2014). Identification of tire forces using Dual Unscented Kalman Filter algorithm. Nonlinear Dyn..

[66] Xin X., Chen J., Zou J. Vehicle state estimation using cubature kalman filter. Proceedings of the 17th International Conference on Computational Science and Engineering.

[67] Jin X., Yin G., Hanif A. Cubature kalman filter-based state estimation for distributed drive electric vehicles. Proceedings of the 35th Chinese Control Conference.

[68] Wei W., Bei S., Zhu K., Zhang L., Wang Y. (2016). Vehicle state and parameter estimation based on adaptive cubature Kalman filter. ICIC Express Lett..

[69] Cheng S., Li L., Chen J. (2017). Fusion algorithm design based on adaptive SCKF and integral correction for side-slip angle observation. IEEE Trans. Ind. Electron..

[70] Sun Y., Chen Q. Joint estimation of states and parameters of vehicle model using cubature kalman filter. Proceedings of the IEEE International Conference on Systems, Man, and Cybernetics.

[71] Li J., Zhang J. (2016). Vehicle sideslip angle estimation based on hybrid Kalman filter. Math. Prob. Eng..

[72] Nishida T., Kogushi W., Takagi N., Kurogi S. Dynamic state estimation using particle filter and adaptive vector quantizer. Proceedings of the IEEE International Symposium on Computational Intelligence in Robotics and Automation.

[73] Wang B., Cheng Q., Victorino A.C., Charara A. Nonlinear observers of tire forces and sideslip angle estimation applied to road safety: Simulation and experimental validation. Proceedings of the 15th International IEEE Conference on Intelligent Transportation Systems.

[74] Chu W., Luo Y., Dai Y., Li K. (2015). In–wheel motor electric vehicle state estimation by using unscented particle filter. Int. J. Veh. Des..

[75] Zhao H., Chen H. Estimation of vehicle yaw rate and side slip angle using moving horizon strategy. Proceedings of the 6th World Congress on Intelligent Control and Automation.

[76] Canale M., Fagiano L., Novara C. A direct Moving Horizon approach to vehicle side-slip angle estimation. Proceedings of the 49th IEEE Conference on Decision and Control.

[77] Strano S., Terzo M. (2017). Vehicle sideslip angle estimation via a Riccati equation based nonlinear filter. Meccanica.

[78] O’Brien R.T., Kiriakidis K.A. Comparison of H_∞_ with Kalman Filtering in Vehicle State and Parameter Identification. Proceedings of the American Control Conference.

[79] Brembeck J. (2019). Nonlinear constrained moving horizon estimation applied to vehicle position estimation. Sensors.

[80] Dawood M., Cappelle C., El Najjar M.E., Khalil M., Pomorski D. Vehicle geo-localization based on IMM-UKF data fusion using a GPS receiver, a video camera and a 3D city model. Proceedings of the IEEE Intelligent Vehicles Symposium.

[81] Arasaratnam I., Haykin S. (2009). Cubature kalman filters. IEEE Trans. Autom. Control.

[82] Tanelli M., Piroddi L., Savaresi S.M. (2009). Real-time identification of tire-road friction conditions. IET Control Theory Appl..

[83] Rajamani R., Phanomchoeng G., Piyabongkarn D., Lew J.Y. (2011). Algorithms for real-time estimation of individual wheel tire-road friction coefficients. IEEE/ASME Trans. Mechatron..

[84] Nam K. (2015). Application of novel lateral tire force sensors to vehicle parameter estimation of electric vehicles. Sensors.

[85] Lian Y.F., Zhao Y., Hu L.L., Tian Y.T. (2015). Cornering stiffness and sideslip angle estimation based on simplified lateral dynamic models for four-in-wheel-motor-driven electric vehicles with lateral tire force information. Int. J. Autom. Technol..

[86] Chen L., Bian M., Luo Y., Qin Z., Li K. (2016). Tire-road friction coefficient estimation based on the resonance frequency of in-wheel motor drive system. Veh. Syst. Dyn..

[87] Choi M., Oh J.J., Choi S.B. (2013). Linearized recursive least squares methods for real-time identification of tire-road friction coefficient. IEEE Trans. Veh. Technol..

[88] Kim C.S., Hahn J.O., Hong K.S., Yoo W.S. (2014). Estimation of tire-road friction based on onboard 6-DoF acceleration measurement. IEEE Trans. Veh. Technol..

[89] Stephant J., Charara A., Meizel D. Linear observers for vehicle sideslip angle: Experimental validation. Proceedings of IEEE International Symposium on Industrial Electronics.

[90] Zhao Y.Q., Li H.Q., Lin F., Wang J., Ji X.W. (2017). Estimation of road friction coefficient in different road conditions based on vehicle braking dynamics. Chin. J. Mech. Eng..

[91] Zhang H., Zhang G., Wang J. (2015). Sideslip Angle Estimation of an Electric Ground Vehicle via Finite-Frequency H_∞_ Approach. IEEE Trans. Transp. Electrif..

[92] Chen T., Chen L., Cai Y., Xu X. (2018). Robust sideslip angle observer with regional stability constraint for an uncertain singular intelligent vehicle system. IET Control Theory Appl..

[93] Ozkan B., Margolis D., Pengov M. (2008). The controller output observer: Estimation of vehicle tire cornering and normal forces. ASME Trans. J. Dyn. Syst. Meas. Control.

[94] Hsiao T. (2012). Robust estimation and control of tire traction forces. IEEE Trans. Veh. Technol..

[95] Ahn C., Peng H., Tseng H.E. (2012). Robust estimation of road friction coefficient using lateral and longitudinal vehicle dynamics. Veh. Syst. Dyn..

[96] Ahn C., Peng H., Tseng H.E. (2011). Robust estimation of road frictional coefficient. IEEE Trans. Control Syst. Technol..

[97] Zhao J., Zhang J., Zhu B. (2016). Coordinative traction control of vehicles based on identification of the tyre-road friction coefficient. Proc. Inst. Mech. Eng. D J. Autom. Eng..

[98] Cadiou J.C., El Hadri A., Chikhi F. (2004). Non-linear tyre forces estimation based on vehicle dynamics observation in a finite time. Proc. Inst. Mech. Eng. D J. Autom. Eng..

[99] Lee D.J., Park Y.S. (2011). Sliding-mode-based parameter identification with application to tire pressure and tire-road friction. Int. J. Autom. Technol..

[100] Song Z.B., Zweiri Y.H., Seneviratne L.D., Althoefer K. (2008). Non-linear observer for slip estimation of tracked vehicles. Proc. Inst. Mech. Eng. D J. Autom. Eng..

[101] Subudhi B., Ge S.S. (2012). Sliding-mode-observer-based adaptive slip ratio control for electric and hybrid vehicles. IEEE Trans. Intell. Transp. Syst..

[102] Patel N., Edwards C., Spurgeon S.K. (2007). Optimal braking and estimation of tyre friction in automotive vehicles using sliding modes. J. Mech. Syst. Sci..

[103] Tanelli M., Ferrara A., Giani P. Combined vehicle velocity and tire-road friction estimation via sliding mode observers. Proceedings of the IEEE International Conference on Control Applications.

[104] M’sirdi N.K., Rabhi A., Fridman L., Davila J., Delanne Y. Second order sliding mode observer for estimation of velocities, wheel sleep, radius and stiffness. Proceedings of the American Control Conference.

[105] Patel N., Edwards C., Spurgeon S.K. (2008). Tyre-road friction estimation—A comparative study. Proc. Inst. Mech. Eng. D J. Autom. Eng..

[106] Khemoudj O., Imine H., Djemai M. (2013). Heavy duty vehicle tyre forces estimation using variable gain sliding mode observer. Int. J. Veh. Des..

[107] Chen Y., Ji Y., Guo K. (2014). A reduced-order nonlinear sliding mode observer for vehicle slip angle and tyre forces. Veh. Syst. Dyn..

[108] Rath J.J., Veluvolu K.C., Defoort M., Soh Y.C. (2014). Higher-order sliding mode observer for estimation of tyre friction in ground vehicles. Iet Control Theory Appl..

[109] Chen T., Chen L., Xu X., Cai Y., Jiang H., Sun X. (2018). Estimation of longitudinal force and sideslip angle for intelligent four-wheel independent drive electric vehicles by observer iteration and information fusion. Sensors.

[110] Imine H., Benallegue A., Madani T., Srairi S. (2013). Rollover risk prediction of heavy vehicle using high-order sliding-mode observer: Experimental results. IEEE Trans. Veh. Technol..

[111] Zhao L.H., Liu Z.Y., Chen H. (2010). Design of a nonlinear observer for vehicle velocity estimation and experiments. IEEE Trans. Control Syst. Technol..

[112] Imsland L., Johansen T.A., Fossen T.I., Grip H.F., Kalkkuhl J.C., Suissa A. (2006). Vehicle velocity estimation using nonlinear observers. Automatica.

[113] Guo H., Chen H., Cao D., Jin W. (2013). Design of a reduced-order non-linear observer for vehicle velocities estimation. Iet Control Theory Appl..

[114] Grip H.F., Imsland L., Johansen T.A., Fossen T.I., Kalkkuhl J.C., Suissa A. (2008). Nonlinear vehicle side-slip estimation with friction adaptation. Automatica.

[115] Gao X., Yu Z., Neubeck J., Wiedemann J. (2010). Sideslip angle estimation based on input-output linearisation with tire-road friction adaptation. Veh. Syst. Dyn..

[116] Solmaz S., Başlamışlı S.Ç. (2012). A nonlinear sideslip observer design methodology for automotive vehicles based on a rational tire model. Int. J. Adv. Manuf. Technol..

[117] Phanomchoeng G., Rajamani R., Piyabongkarn D. (2011). Nonlinear observer for bounded Jacobian systems, with applications to automotive slip angle estimation. IEEE Trans. Autom. Control.

[118] Li L., Song J., Kong L., Huang Q. (2009). Vehicle velocity estimation for real-time dynamic stability control. Int. J. Autom. Technol..

[119] Chen C., Jia Y., Wang Y., Shu M. (2018). Non-linear velocity observer for vehicles with tyre–road friction estimation. Int. J. Syst. Sci..

[120] Hashemi E., Zarringhalam R., Khajepour A., Melek W., Kasaiezadeh A., Chen S.K. (2017). Real-time estimation of the road bank and grade angles with unknown input observers. Veh. Syst. Dyn..

[121] Stéphant J., Charara A. Observability matrix and parameter identification: Application to vehicle tire cornering stiffness. Proceedings of the 44th IEEE Conference on Decision and Control.

[122] Sun F., Huang X., Rudolph J., Lolenko K. (2015). Vehicle state estimation for anti-lock control with nonlinear observer. Control Eng. Pr..

[123] Solmaz S., Başlamışlı S.Ç. (2012). Simultaneous estimation of road friction and sideslip angle based on switched multiple non-linear observers. IET Control Theory Appl..

[124] Ko S.Y., Ko J.W., Lee S.M., Cheon J.S., Kim H.S. (2014). Vehicle velocity estimation using effective inertia for an in-wheel electric vehicle. Int. J. Autom. Technol..

[125] Xia X., Xiong L., Sun K., Yu Z.P. (2016). Estimation of maximum road friction coefficient based on Lyapunov method. Int. J. Autom. Technol..

[126] Wang R., Wang J. (2013). Tire-road friction coefficient and tire cornering stiffness estimation based on longitudinal tire force difference generation. Control Eng. Pr..

[127] Erdogan G., Alexander L., Rajamani R. (2010). Estimation of tire-road friction coefficient using a novel wireless piezoelectric tire sensor. IEEE Sens. J..

[128] Hong S., Erdogan G., Hedrick K., Borrelli F. (2013). Tyre-road friction coefficient estimation based on tyre sensors and lateral tyre deflection: Modelling, simulations and experiments. Veh. Syst. Dyn..

[129] Zhang J., Wang F.Y., Wang K., Lin W.H., Xu X., Chen C. (2011). Data-driven intelligent transportation systems: A survey. IEEE Trans. Intell. Transp. Syst..

[130] Wei Y., Zhang X., Shi Y., Xia L., Pan S., Wu J., Zhao X. (2018). A review of data-driven approaches for prediction and classification of building energy consumption. Renew. Sust. Energ. Rev..

[131] You G.W., Park S., Oh D. (2016). Real-time state-of-health estimation for electric vehicle batteries: A data-driven approach. Appl. Energy.

[132] Gurney K. (2004). An Introduction to Neural Networks.

[133] Dong G., Zhang X., Zhang C., Chen Z. (2015). A method for state of energy estimation of lithium-ion batteries based on neural network model. Energy.

[134] Chang Y., Jiang T., Pu Z. (2017). Adaptive control of hypersonic vehicles based on characteristic models with fuzzy neural network estimators. Aerosp. Sci. Technol..

[135] Shafiei M.H., Binazadeh T. (2015). Application of neural network and genetic algorithm in identification of a model of a variable mass underwater vehicle. Ocean. Eng..

[136] Hatamleh K.S., Al-Shabi M., Al-Ghasem A., Asad A.A. (2015). Unmanned aerial vehicles parameter estimation using artificial neural networks and iterative bi-section shooting method. Appl. Soft. Comput..

[137] Saadeddin K., Abdel-Hafez M.F., Jaradat M.A., Jarrah M.A. (2013). Performance enhancement of low-cost, high-accuracy, state estimation for vehicle collision prevention system using ANFIS. Mech. Syst. Signal Process..

[138] Vargas-Melendez L., Boada B., Boada M., Gauchia A., Diaz V. (2017). Sensor Fusion Based on an Integrated Neural Network and Probability Density Function (PDF) Dual Kalman Filter for On-Line Estimation of Vehicle Parameters and States. Sensors.

[139] Nguyen M.-H., Zhou C. (2010). Improving GPS/INS integration through neural networks. arXiv.

[140] Gwak M., Jo K., Sunwoo M. (2013). Neural-network multiple models filter (NMM)-based position estimation system for autonomous vehicles. Int. J. Autom. Technol..

[141] Kim H.U., Bae T.S. (2019). Deep Learning-Based GNSS Network-Based Real-Time Kinematic Improvement for Autonomous Ground Vehicle Navigation. J. Sens..

[142] Boada B.L., Boada M.J.L., Gauchía A., Olmeda E., Díaz V. (2015). Sideslip angle estimator based on ANFIS for vehicle handling and stability. J. Mech. Sci. Technol..

[143] Boada B.L., Boada M.J.L., Diaz V. (2016). Vehicle sideslip angle measurement based on sensor data fusion using an integrated ANFIS and an Unscented Kalman Filter algorithm. Mech. Syst. Signal Process..

[144] Wei W., Bei S., Zhang L., Zhu K., Wang Y., Hang W. (2016). Vehicle sideslip angle estimation based on general regression neural network. Math. Prob. Eng..

[145] Liu H., Yang J., Yang H., Yi F. (2016). Soft sensor of vehicle state estimation based on the kernel principal component and improved neural network. J. Sens..

[146] Melzi S., Sabbioni E. (2011). On the vehicle sideslip angle estimation through neural networks: Numerical and experimental results. Mech. Syst. Signal Process..

[147] Ghosh J., Tonoli A., Amati N. (2018). A Deep Learning based Virtual Sensor for Vehicle Sideslip Angle Estimation: Experimental Results. SAE Tech. Pap..

[148] Boada B.L., Boada M.J.L., Vargas-Melendez L., Diaz V. (2018). A robust observer based on H∞ filtering with parameter uncertainties combined with Neural Networks for estimation of vehicle roll angle. Mech. Syst. Signal Process..

[149] García Guzmán J., Prieto González L., Pajares Redondo J., Montalvo Martínez M.L., Boada M. (2018). Real-Time Vehicle Roll Angle Estimation Based on Neural Networks in IoT Low-Cost Devices. Sensors.

[150] Vargas-Meléndez L., Boada B., Boada M., Gauchía A., Díaz V. (2016). A sensor fusion method based on an integrated neural network and Kalman filter for vehicle roll angle estimation. Sensors.

[151] Acosta M., Kanarachos S. (2018). Tire lateral force estimation and grip potential identification using Neural Networks, Extended Kalman Filter, and Recursive Least Squares. Neural. Comput. Appl..

[152] Matuško J., Petrović I., Perić N. (2008). Neural network based tire/road friction force estimation. Eng. Appl. Artif Intel..

[153] Xu D., Yap F.F., Han X., Wen G.L. (2003). Identification of spring-force factors of suspension systems using progressive neural network on a validated computer model. Inverse. Probl. Sci. Eng..

[154] Dye J., Lankarani H. Hybrid simulation of a dynamic multibody vehicle suspension system using neural network modeling fit of tire data. Proceedings of the ASME Design Engineering Technical Conference.

[155] Alagappan A.V., Rao K.N., Kumar R.K. (2015). A comparison of various algorithms to extract Magic Formula tyre model coefficients for vehicle dynamics simulations. Veh. Syst. Dyn..

[156] Song S., Min K., Park J., Kim H., Huh K. Estimating the Maximum Road Friction Coefficient with Uncertainty Using Deep Learning. Proceedings of the 21st International Conference on Intelligent Transportation Systems.

[157] Castillo Aguilar J., Cabrera Carrillo J., Guerra Fernández A., Carabias Acosta E. (2015). Robust road condition detection system using in-vehicle standard sensors. Sensors.

[158] Zareian A., Azadi S., Kazemi R. (2016). Estimation of road friction coefficient using extended Kalman filter, recursive least square, and neural network. Proc. Inst. Mech. Eng. K J. Mul. Dyn..

[159] Liu J., Cheng K.W.E., Zeng J. (2015). A novel multi-sensors fusion framework based on Kalman Filter and neural network for AFS application. T.I. Meas.Control..

[160] Taghavifar H., Mardani A. (2014). Use of artificial neural networks for estimation of agricultural wheel traction force in soil bin. Neural. Comput. Appl..

[161] Yousefzadeh M., Azadi S., Soltani A. (2010). Road profile estimation using neural network algorithm. J. Mech. Sci. Technol..

[162] Solhmirzaei A., Azadi S., Kazemi R. (2012). Road profile estimation using wavelet neural network and 7-DOF vehicle dynamic systems. J. Mech. Sci. Technol..

[163] Luque P., Mántaras D.A., Fidalgo E., álvarez J., Riva P., Girón P., Ferran J. (2013). Tyre-road grip coefficient assessment—Part II: Online estimation using instrumented vehicle, extended Kalman filter, and neural network. Veh. Syst. Dyn..

[164] Wang L., Zhang N., Du H. (2015). Real-time identification of vehicle motion-modes using neural networks. Mech. Syst. Signal Process..

[165] Yu R., Xia X. (2015). Vehicle handling evaluation models using artificial neural networks. Int. J. Control Autom..

[166] Wefky A.M., Espinosa F., Jiménez J.A., Santiso E., Rodríguez J.M., Fernández A.J. (2010). Alternative sensor system and MLP neural network for vehicle pedal activity estimation. Sensors.

[167] Li L., Qian B., Lian J., Zheng W., Zhou Y. (2017). Traffic scene segmentation based on RGB-D image and deep learning. IEEE Trans. Intell. Transp. Syst..

[168] Zhang X., Sun J., Qi X., Sun J. (2019). Simultaneous modeling of car-following and lane-changing behaviors using deep learning. Transp. Res. C Emerg. Technol..

